# Letter from Vienna

**Published:** 1868-05-15

**Authors:** Samuel Cole

**Affiliations:** Vienna


### *FOREIGN CORRESPONDENCE.*

VIENNA, *April* 8*th*, 1868.

*To the Editor of the Chicago Medical Journal:*

DEAR SIR,— After a prolonged sojourn in Heidelberg, I took advantage of an offer made to me by Dr. Knapp, Professor of Ophthalmology in that city, to accompany him on a scientific tour through several of the principal cities of Germany.

Our first stopping place was Munich, where we visited one of the most celebrated opticians of Europe, Mr. Steinheil. His institution enjoys the highest reputation for accuracy of manufacture of perfectly aplanatic dioptrical objectives, of prisms, and of glass plates with plane and parallel surfaces. To fabricate these latter without a trace of astigmatism, *i. e.*, without any irregularity of surface, or inequality of refractive power, is recognized as one of the most difficult tasks of practical optics.

Selecting from many interesting things, I may mention two applications of optics to practical ophthalmology, one consisting of an easy method of expressing the various degrees of refractive and accommodative power by the size of angles instead of the inverse value of their focal distances (*i. e.*, 1 divided by the focal distance of the lens). This method renders the calculation much simpler by substituting integral for fractional numbers. On the other hand, it appears less convenient for practical purposes since the degree of hyperopia or myopia is expressed in a way different from that of the visual distance of the eye. To render this clearer, I must resort to an illustration. If a ray of light coming from a luminous point fall upon a lens at a certain point above its axis, the refractive power of this lens may be measured by the angle of deviation which the refracted ray undergoes. The higher the refractive power of the lens, the greater will be the angle of deviation. Mr. Steinheil found by calculation that for a given and very small *height of incidence* (distance of the point of incidence from the axis) the angles of deviation for different lenses, the luminous point remaining stationary, are as follows:

Refractive Power of Lens Expressed

| By angle of deviation in seconds. | By focal distance in inches. | By angle of deviation in seconds. | By focal distance in inches. |
|---|---|---|---|
| 1 | 120. | 15 | 8. |
| 2 | 60. | 20 | 6. |
| 3 | 40. | 24 | 5. |
| 4 | 30. | 30 | 4. |
| 5 | 24. | 40 | 3. |
| 6 | 30. | 48 | 2.5 |
| 7 | 17.14 | 50 | 2.4 |
| 8 | 15. | 60 | 2. |
| 9 | 13.33 | 80 | 1.5 |
| 10 | 12. | 100 | 1.2 |
| 11 | 10.91 | 120 | 1. |
| 12 | 10. | | |

By this table you will perceive that the refractive power of a lens of $\frac{1}{120}$ is equal to an angle of deviation of 1″; consequently an augmentation of refractive power of $\frac{1}{120}$ increases the size of the angle of deviation by 1″. If, for instance, a short-sighted person can see plainly at eight inches distance, modern ophthalmologists say that the grade of his myopia is $\frac{1}{8}$. According to Mr. Steinheil, we would say that is equivalent to an angle of deviation of 15″. The method in use expresses the visual distance and the degree of myopia in the same manner, whilst Steinheil's has the inconvenience that the degree of myopia is expressed by an angle, the visual distance being, of necessity, expressed by its length.

As it is probable that both methods will be used, and an easy reduction from one to the other can be made, I thought it useful to send the foregoing table to you.

Another interesting object was a peculiar lens, constructed on the terrestrian telescopes, for the use of very short-sighted persons. The apparatus is a truncated glass cone, three-fourths of an inch in length, its diameter at the middle, one third of an inch, its broader extremity convex, its smaller, concave. Aside from its portability it has the advantage, when its convex surface is directed at distant objects, of magnifying slightly. When we consider that the ordinary strong concave glasses, such as are required by the higher grades of myopia in order to see distant objects with distinctness, diminish their apparent size so considerably as to render them almost useless, we can conceive what benefit can be derived from this combination of convex and concave surfaces.

We next visited Prof. Rothmund's Ophthalmic Hospital, containing about forty beds, and there had an opportunity of seeing a number of cases of a peculiar (hitherto undescribed) skin disease, subsequently complicated with cataract. They all were of children coming from a village, situated in a valley of the Bavarian Alps. This village is rather isolated, consequently marriages among kindred very frequent.

During the first months the children appeared healthy. At the fourth month, or somewhat later, the integument of the face and extremities became thickened, and covered with red patches, whilst the trunk itself remained unaffected. On microscopical examination, the disease proves to be a dermatitis, with hypertrophy of chorion and epithelial layers, the most remarkable characteristic of which is, that as the affection spreads, both crystalline lenses become cloudy, and in the course of a few weeks, completely milky and opaque. It is the ordinary soft cataract, and can be operated (discission) with favorable prognosis. That these cataracts should occur as complications to a dermatitis is a highly interesting sequence of the fact, so well known in embryology, that the lens is formed by inversion of a portion of the skin, both tissues, so different in ultimate structure but of common origin, undergoing the same pathological changes.

This coincidence would have been utterly inexplicable if the development of the lens from the epithelial layer of the fœtal integuments were not known.

And now, my dear sir, I fear that it may not be so interesting for you to read, as for me to write these descriptions, and therefore beg leave to sign myself,

Yours, truly, DR. SAMUEL COLE.

